# A Versatile Plasmid System for Translational Control and Secretion of Recombinant Proteins in Mycobacteria

**DOI:** 10.1021/acssynbio.5c00966

**Published:** 2026-01-21

**Authors:** Victor Gigante Pereira, Paloma Rezende Corrêa, Rodrigo Martins Barros, Meydson Benjamim Carvalho Corrêa, Odir Antonio Dellagostin, Leila Mendonça-Lima, Marcos Gustavo Araujo Schwarz

**Affiliations:** † Laboratório de Biologia Molecular Aplicada à Micobactérias, Instituto Oswaldo Cruz, 37903 Fiocruz, Rio de Janeiro, Rio de Janeiro 21040-900, Brazil; ‡ Núcleo de Biotecnologia, Centro de Desenvolvimento Tecnológico, 564099 Universidade Federal de Pelotas, Pelotas, Rio Grande do Sul 96010-610, Brazil

**Keywords:** mycobacteria, riboswitch, protein secretion, translational regulation, recombinant expression, synthetic biology

## Abstract

Recombinant protein expression in mycobacteria faces two major challenges: limited regulatory tools for inducible expression and inefficient secretion of heterologous products. In this study, we developed plasmid-based systems that enable translationally gated secretion in *Mycobacterium smegmatis*, coupling riboswitch-mediated translational control with efficient extracellular export. The platform integrates the *M. tuberculosis* antigen 85A promoter and signal peptide for constitutive secretion combined with synthetic riboswitches for inducible translational regulation. We tested two theophylline-responsive riboswitches (riboE and riboE+) and a temperature-sensitive variant (riboU9) by using mCherry as a reporter. Fluorescence assays, RT-PCR, and Western blotting confirmed efficient secretion and strict translational control. The theophylline-inducible systems exhibited a dose-dependent response with maximal expression at 2 mM inducer, while the riboU9 construct showed a clean ON/OFF phenotype triggered by temperature shift. In all cases, transcripts were detected irrespective of induction, confirming regulation at the translational rather than transcriptional level. Secretion was highly efficient, with 10–20 fold higher protein levels in extracellular versus intracellular fractions. Induction during early- and mid-log phases yielded maximal protein, whereas late-log induction reduced output by ∼50%. Together, these results define translationally gated secretion as a new control layer in mycobacterial protein production. This modular platform expands the genetic toolkit available for Mycobacterium research, providing new opportunities for the study of antigens and virulence factors from slow-growing pathogens and offering potential applications in structural biology, vaccine development, and drug target validation.

## Graphical abstract

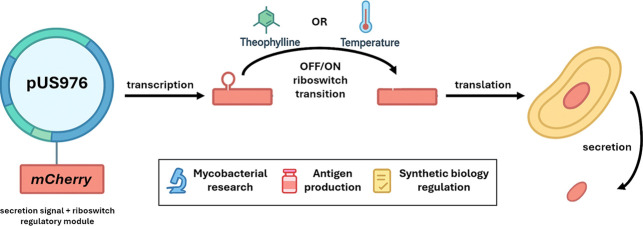

## 1 Introduction

Recombinant DNA technology has revolutionized biological research and propelled biotechnology to new levels. Among its many applications, the genetic engineering of host organisms for recombinant protein expression—often through heterologous systems—remains a cornerstone of molecular biology.^1^ The Gram-negative bacterium *Escherichia coli* continues to be the predominant host due to its well-characterized genetics, inexpensive culture requirements, and rapid doubling time. Over the years, *E. coli* has been extensively optimized as a protein production chassis through strategies aimed at improving expression levels, codon usage, tRNA availability, plasmid stability, and even protein folding efficiency.^2^

The expression system choice, however, depends on the properties of the target protein and the intended application. For proteins requiring complex folding or post-translational modifications—particularly therapeutic biologics—eukaryotic systems such as mammalian, yeast, or insect cells are often preferred, as they better reproduce the native biosynthetic environment. Microbial platforms, nevertheless, retain significant advantages in terms of scalability, cost-effectiveness, and throughput.^3^

In the context of mycobacterial research, heterologous expression has been transformative. The slow growth of clinically relevant species such as *Mycobacterium tuberculosis* and the inability to culture others such as *M. leprae* severely limit direct protein studies.^4^ By expressing mycobacterial proteins in tractable hosts—*E. coli* or fast-growing mycobacteria like *M. smegmatis*—researchers can bypass these constraints, accelerating studies on virulence factors, drug targets, and immune evasion strategies. However, heterologous expression of mycobacterial proteins in *E. coli* remains challenging, with soluble production achieved in fewer than 30% of attempts, even under optimized conditions.^5,6^ Inefficient folding, aggregation, secretion bottlenecks, low expression yields, and frequent inclusion and body formation are major obstacles. In addition, differences in codon usage and the high GC content of mycobacterial genomes further compromise expression efficiency.^7^ These challenges highlight the importance of designing specialized expression systems tailored to mycobacteria.

Another critical factor for successful protein production is the precise regulation of expression, ideally synchronized with optimal host growth phases to minimize metabolic burden. Most commercial expression systems rely on transcriptional regulation, such as the lac and ara operons in *E. coli*.^8,9^ In mycobacteria, fewer options are available, with inducible systems including the acetamidase promoter and TetR-based platforms responsive to nonantibiotic inducers like anhydrotetracycline.^10^ Although effective, these systems require protein regulators, adding metabolic cost and complexity.^11^

Riboswitches provide a versatile alternative, functioning mainly through translational control.^12^ Located in the 5′ untranslated region (UTR) of mRNA, they undergo conformational changes upon effector binding that modulate ribosome access to the start codon.^13^ Unlike protein-based regulators, riboswitches act autonomously and do not require additional factors for activity. Structurally, they consist of two modules: an aptamer domain that binds the effector molecule and an expression platform containing the ribosome binding site (RBS) and start codon. Binding induces conformational changes that expose the RBS (ON state) or occlude it (OFF state).^14^ Natural examples include the vitamin B_12_-responsive riboswitch in *M. tuberculosis*, which regulates resuscitation-promoting factors critical for persistence and reactivation.^15^ Synthetic designs, such as the theophylline-responsive riboswitch and thermosensitive variants, have further demonstrated the versatility of RNA-based regulatory systems.^16^

A strategy that greatly facilitates downstream processing in recombinant protein production is the use of secretion systems capable of exporting the final product directly into the culture medium, thereby exploiting the endogenous export machinery of the host organism. In this context, the Antigen 85A (Ag85A) secretion system from *M. tuberculosis* represents an effective model. Ag85A is a major secreted protein that carries a canonical N-terminal signal peptide,^17^ which targets the preprotein to the general Sec secretion pathway. Notably, Ag85A secretion appears to be independent of specialized accessory pathways such as SecA2,^18,19^ as it has not been identified among the proteins whose secretion is affected in *M. tuberculosis* *secA2* knockout strains.^20^ Mechanistically, the ATPase SecA recognizes the signal peptide and, through successive cycles of ATP hydrolysis, drives the translocation of the unfolded preprotein across the cytoplasmic membrane via the SecYEG channel.^21^ Harnessing this native secretion pathway enables the direct recovery of a properly processed and soluble recombinant protein from the culture supernatant, thereby simplifying purification workflows.

Despite significant progress in regulated gene expression systems for mycobacteria, current platforms largely focus on transcriptional control and implicitly assume that secretion is a passive downstream consequence of mRNA production. As a result, once transcription is initiated, protein export proceeds constitutively, limiting the ability to synchronize secretion with specific physiological states, growth phases, or experimental conditions. To date, strategies that allow secretion itself to be conditionally permitted or restricted in mycobacteria remain largely unexplored.

In this study, we developed plasmid-based systems for recombinant protein expression in mycobacteria using *M. smegmatis* as a model that enables conditional access to the mycobacterial secretory pathway through translational regulation. To facilitate protein purification, the system was engineered for extracellular secretion by incorporating the promoter and signal peptide of Ag85A, establishing a robust constitutive expression platform. For inducible regulation, we implemented synthetic riboswitches responsive to either theophylline^22,23^ or temperature.^24^ Our results demonstrate efficient extracellular secretion and strict translational-level control in riboswitch-containing constructs with no evidence of transcriptional regulation. The theophylline system displayed a dose-dependent response, while the thermoswitch provided a clear ON/OFF regulation in response to temperature shifts. Together, these findings provide versatile tools for mycobacterial protein research, addressing persistent bottlenecks in expression and purification and expanding the genetic toolkit for functional studies of proteins from slow-growing pathogenic mycobacteria.

## 2 Results

### 2.1 Expression of the Reporter Protein mCherry Using the Constructed Plasmid Variants

To investigate whether secretion in mycobacteria can be conditionally controlled at the translational level, we engineered a set of plasmid variants derived from shuttle vector pUS976. The original construct supports constitutive expression and secretion via the *M. tuberculosis* Ag85A promoter and signal peptide. Riboswitch-regulated derivatives were generated by inserting synthetic translational control elements upstream of the signal peptide, thereby removing the native ribosome binding site and placing secretion under exclusive riboswitch control (Figure 1). This design enables a direct assessment of whether protein export can be gated independently of transcription.

**Figure 1:**
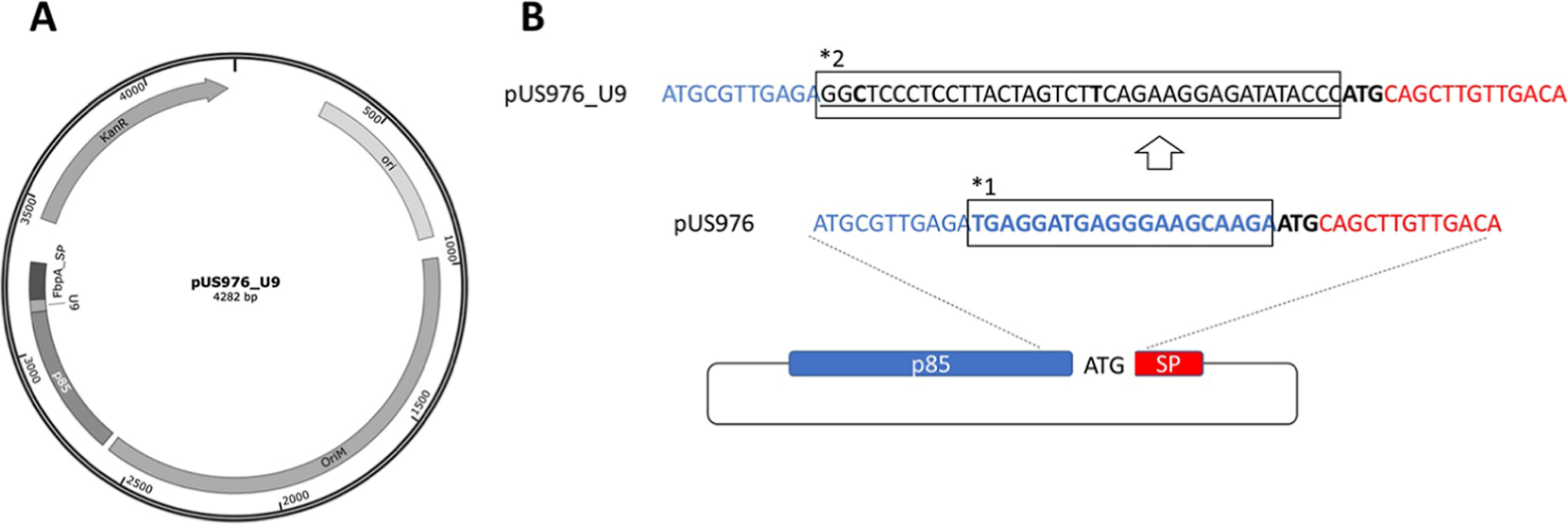
Plasmid design with integrated riboswitches. (A) Map of the shuttle vector pUS976_U9, featuring replication origins for *E. coli* (ori) and mycobacteria (OriM), a kanamycin resistance marker (KanR), and an expression cassette under the *M. tuberculosis* antigen 85 promoter (p85), regulated by the U9 thermoswitch and directed for secretion via the FbpA signal peptide (SP). (B) Strategy for riboswitch insertion, exemplified with pUS976_U9: the native 3′-terminal 20 nt (boxed, *1) of the p85 promoter (blue) in pUS976 were replaced with the riboswitch sequence (underlined, boxed, *2), positioning it upstream of the signal peptide ATG (red). This excision removed the native RBS, ensuring exclusive reliance on the riboswitch-derived RBS for translation initiation.

Functional characterization of the plasmid constructs was carried out to assess reporter gene expression, secretion efficiency, and regulatory control. We also investigated whether regulation occurred at the transcription or translational level. For the theophylline-responsive riboswitches, riboE and riboE+ were used. As previously noted, riboE + constitutes a modified version of riboE, optimized for application in Gram-positive bacteria. This adaptation addresses the characteristically shorter RBS-to-start-codon distance found in species such as *M. tuberculosis*.^25^ Within the established literature, riboE + exhibited a reduced level of basal expression; however, this is accompanied by a more constrained dynamic range in comparison to the original riboE construct.^22^ With these constructs, a dose–response assay was first performed to determine the minimal inducer concentration required for maximal expression. As shown in Figure 2, high fluorescence was obtained as early as 2 mM theophylline, peaking at 3 mM, as previously described for this riboswitch in mycobacteria,^23^ which was therefore selected for all subsequent experiments with these constructs.

**Figure 2:**
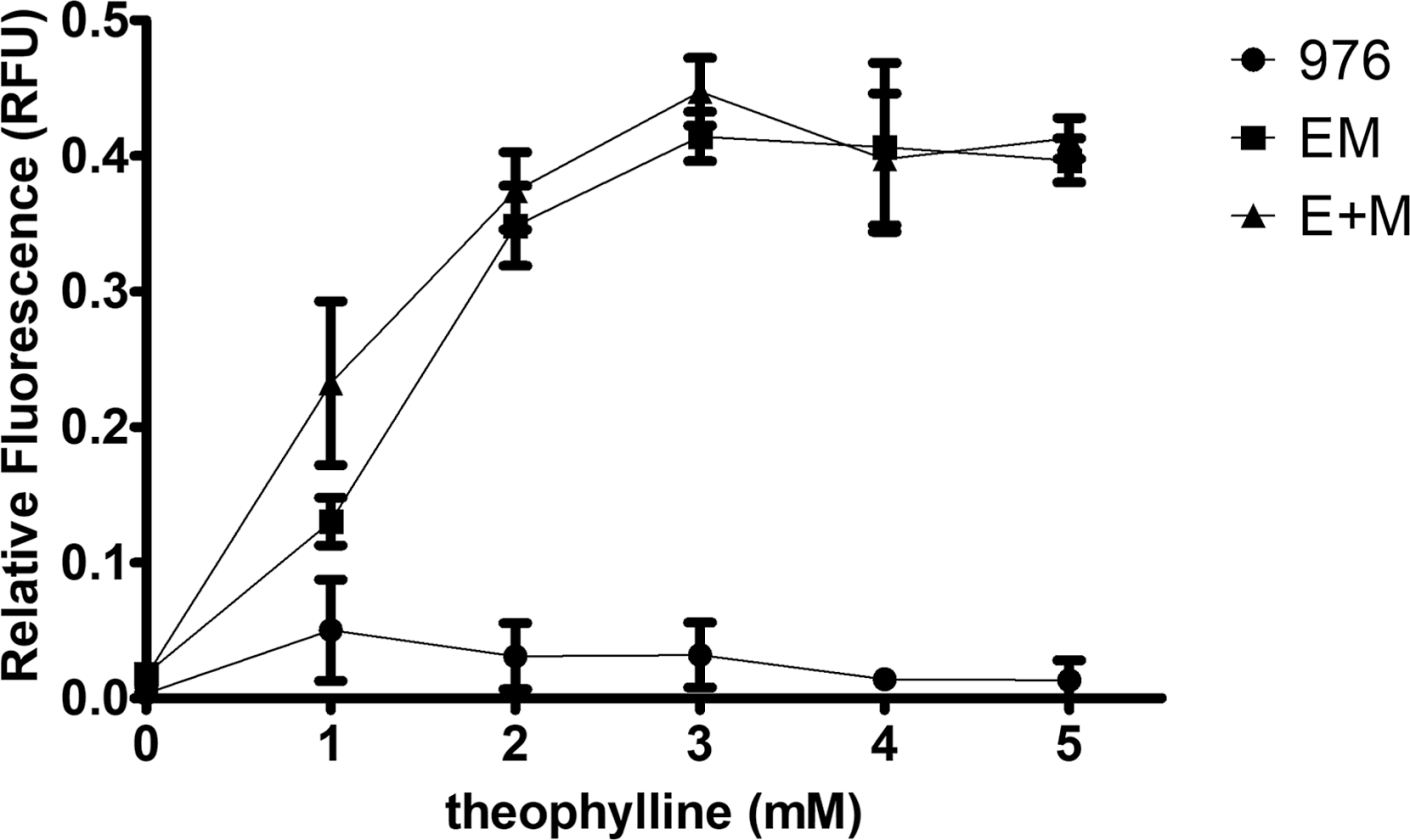
Theophylline dose–response curve in recombinant *M. smegmatis mc* ^2^ 155 strains. Relative fluorescence units (RFU) were measured from secreted fractions of strains carrying the empty pUS976 vector (negative control, 976), riboE–mCherry construct (EM), or riboE+–mCherry construct (E + M), cultured with increasing concentrations of theophylline (0–5 mM). Data represent the mean ± SD of three biological replicates.

In addition to high expression levels, the engineered system successfully secreted the reporter protein, as demonstrated by the detection of fluorescence in the extracellular fraction. Fluorimetric assays confirmed the specificity of this signal: cultures harboring the empty pUS976 vector showed no significant RFU above the background. These results validate both the secretion capability of the platform and the reliability of mCherry as a reporter under the tested conditions.

Reporter gene expression was next analyzed across all constructs under both induced and noninduced conditions. For theophylline-responsive riboswitches (riboE and riboE+), induction was performed with 2 mM theophylline, whereas for riboU9, the thermoswitch variant, expression was tested at 20 °C, 25 °C, and 37 °C. As shown in Figure 3, transcriptional analysis detected *mCherry* transcripts in all strains carrying the reporter gene, with the expected exception of the mc^2^ control strain harboring the empty pUS976 plasmid. Notably, transcript production persisted under both induced and noninduced conditions, suggesting that expression regulation does not occur at the transcriptional level, as cellular machinery continued to produce the mRNA irrespective of induction state.

**Figure 3:**
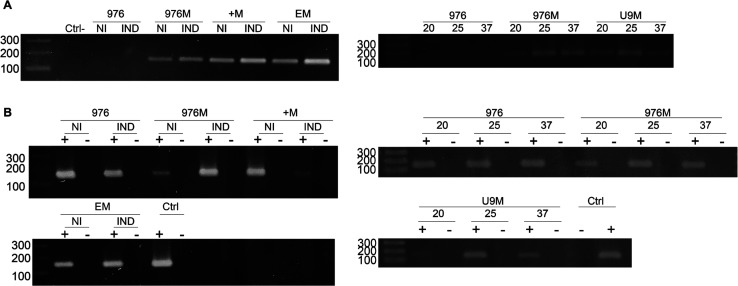
Transcriptional analysis of riboswitch-regulated mCherry expression in *M. smegmatis*. (A) *mCherry* and (B) *mysA* transcripts detected by RT-PCR in strains carrying empty pUS976 (976), constitutive 976–mCherry (976M), theophylline-inducible riboE+–mCherry (+M) and riboE–mCherry (EM), and temperature-sensitive riboU9–mCherry (U9). Induction with 2 mM theophylline was tested for riboE/E+ constructs (noninduced [NI] vs induced [IND]), whereas riboU9 constructs were evaluated at 20 °C, 25 °C, and 37 °C. To confirm RNA purity, samples were subjected to PCR either with (+) or without (−) prior reverse transcriptase treatment following DNase digestion. Genomic DNA and no–template reactions served as positive and negative controls, respectively.

Functional characterization of the riboswitch-regulated system demonstrated strict translational control of the mCherry production. Both fluorimetric analysis (Figure 4) and Western blotting (Figure 5) showed that fluorescence and the full-length polypeptide were detected only under induced conditions in riboswitch-containing constructs. The temperature-sensitive riboU9 variant displayed particularly strong regulation: reporter protein was absent at the nonpermissive temperature (20 °C), but a shift to 25 or 37 °C activated the riboswitch and triggered translation. Together with the persistent detection of mCherry transcripts under all conditions (Figure 3), these results provide clear evidence that regulation occurs at the translational rather than transcriptional level.

**Figure 4:**
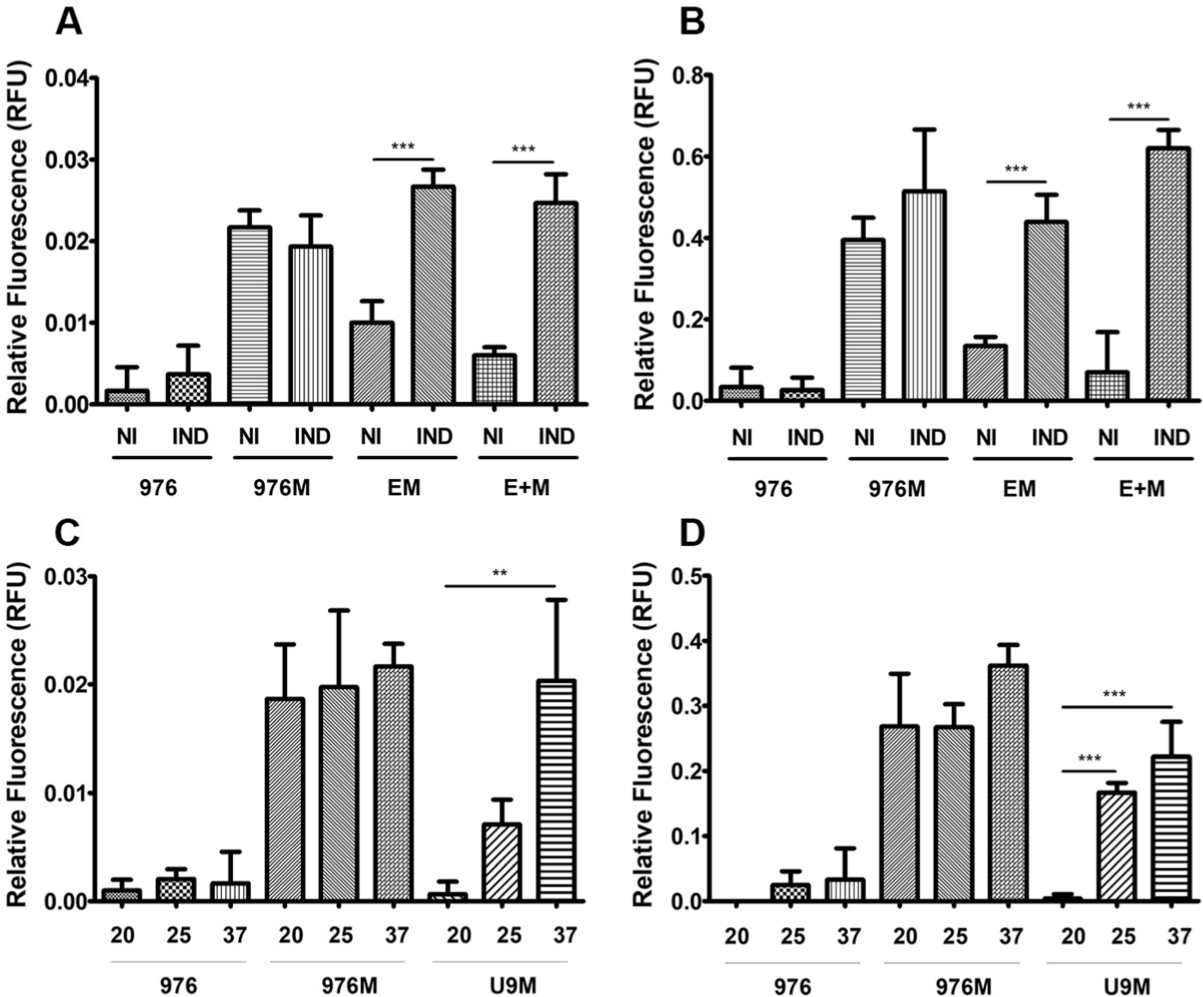
Fluorescence detection of mCherry in recombinant *M. smegmatis* strains under induced and noninduced conditions. Relative fluorescence units (RFU) were measured (excitation: 587 nm, emission: 610 nm, bandwidth: 10 nm) using a microplate reader in (A,C) intracellular and (B,D) secreted fractions. Strains analyzed: empty pUS976 (976), constitutive 976–mCherry (976M), theophylline-inducible riboE+–mCherry (+M) and riboE–mCherry (EM), and temperature-sensitive riboU9–mCherry (U9). Theophylline induction (2 mM) was applied to riboE/E+ constructs (noninduced [NI] vs induced [IND]), whereas riboU9 constructs were tested at 20 °C, 25 °C, and 37 °C. Statistical significance: **p < 0.01; ***p < 0.001. Data represent the mean ± SD of three biological replicates.

**Figure 5:**
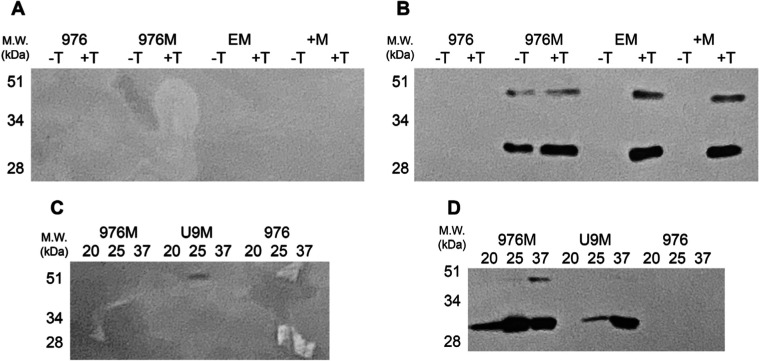
Western blot analysis of mCherry expression in recombinant *M. smegmatis* strains. Protein samples from (A,C) intracellular and (B,D) secreted fractions were resolved by 15% SDS-PAGE and probed with a polyclonal anti-mCherry antibody. Strains analyzed: empty pUS976 (976; negative control), constitutive 976–mCherry (976M; positive control), theophylline-inducible riboE+–mCherry (+M) and riboE–mCherry (EM), and temperature-sensitive riboU9–mCherry (U9). Theophylline induction (2 mM) was tested for riboE/E+ constructs (noninduced [−T] vs induced [+T]), while riboU9 constructs were evaluated at 20 °C, 25 °C, and 37 °C. Molecular weight markers (kDa) are indicated on the left.

The system also exhibited a high secretion efficiency. Western blotting of intracellular fractions revealed no detectable mCherry bands, while fluorimetric assays showed only 5–10% residual signal in cell lysates compared to secreted fractions—a 10–20 fold difference. This combination of efficient secretion and tight translational regulation establishes the engineered platform as a robust tool for the controlled protein production in mycobacteria. The defined induction parameters (2 mM theophylline for riboE/E+ and temperature upshift for riboU9) and the clear ON/OFF behavior make these constructs particularly suitable for applications requiring precise temporal control of heterologous expression.

### 2.2 Expression Induction Test at Different Bacterial Growth Stages

Standardization of the expression system required evaluation of induction dynamics at different stages of bacterial growth (Figure 6). For all riboswitch-containing constructs, mCherry production was similar when induction occurred during early- or mid-log phases. In contrast, induction at the late-log phase resulted in only ∼50% of the RFU obtained at earlier stages. In the temperature-inducible riboU9 system, the onset of fluorescence was delayed compared to the theophylline-inducible constructs (riboE/E+), likely reflecting the physiological adjustment needed after shifting cultures from 20 to 37 °C. Overall, the comparable performance across constructs (except for late-log induction) highlights the robustness of riboswitch-mediated regulation and underscores the importance of growth-phase timing for maximizing protein yield.

**Figure 6:**
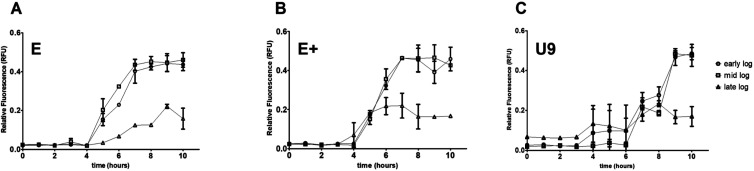
Time-course analysis of riboswitch-mediated induction in recombinant *M. smegmatis*strains. Relative fluorescence units (RFU) were measured hourly after induction of cultures carrying (A) riboE–mCherry, (B) riboE+–mCherry, or (C) riboU9–mCherry constructs. Induction was performed at early-, mid-, and late-log phases. Theophylline (2 mM) was used for riboE/E+ constructs, while riboU9 was activated by a temperature shift to 37 °C. Data represent the mean ± SD of three biological replicates.

## 3 Discussion

In this work, we demonstrate that protein secretion in mycobacteria can be actively gated at the translational level rather than occurring as an inevitable consequence of transcription. By integrating riboswitch-mediated translational control with the Ag85A secretion signal, we establish a system in which mRNA production is uncoupled from protein export and secretion is conditionally permitted only under defined environmental or chemical cues. This represents a conceptual shift from traditional mycobacterial expression platforms in which secretion is constitutively permissive once transcription is initiated.

Efficient secretion remains a critical bottleneck in mycobacterial biotechnology, as many proteins tend to accumulate in insoluble intracellular fractions, often requiring harsh refolding steps.^5^ By redirecting recombinant proteins to the extracellular milieu, our system simplifies downstream purification and reduces cytotoxicity.^20^ With that, secretion-based approaches may accelerate vaccine development and antigen discovery, particularly for *M. tuberculosis* proteins, which are otherwise difficult to obtain in functional form. Our results confirm that the 85A signal peptide provides a robust and versatile tool for this purpose.

The regulatory performance of riboswitches was equally noteworthy. Both theophylline-responsive riboE/riboE + constructs and the thermosensitive riboU9 variant achieved tight ON/OFF control, with mCherry expression restricted to induced conditions despite the persistent presence of transcripts. These findings reinforce the role of riboswitches as effective translational regulators, consistent with recent advances in synthetic biology that highlight their modularity and independence from protein-based repressors.^26^ Importantly, the riboU9 system demonstrated a particularly clean phenotype, relying on a temperature shift as a trigger. This provides an alternative to chemical inducers, which may be costly, unstable, or interfere with downstream applications.^27^

The key contribution of this system lies not in the individual genetic components employed but in their functional integration to create translationally gated secretion. In this configuration, the riboswitch acts as a molecular checkpoint that determines whether access to the secretory pathway is granted, independent of transcriptional activity. This architecture enables precise temporal coordination of export and provides a regulatory layer that is orthogonal to classical transcription factor-based systems.

Compared with classical inducible systems in mycobacteria, such as the acetamidase promoter^28^ and TetR-based regulation,^11^ riboswitches offer several advantages: they are genetically compact, do not require accessory proteins, and enable direct coupling of gene expression to environmental or metabolic cues.^29^ A recent review on regulated expression systems in mycobacteria stresses the need for precisely tunable and low-burden alternatives to transcription factor-based control, highlighting riboswitches as a promising solution.^30^ Our results align with this trend and extend it by demonstrating their successful integration with secretion pathways.

An additional outcome of our study was the identification of optimal induction windows. Early- and mid-log induction yielded maximal protein levels, while late-log induction resulted in ∼50% reduced output. This observation is consistent with reports that the growth phase strongly influences recombinant protein production in bacteria, as metabolic activity and translational capacity decline in late-log/early stationary phases.^31,32^ For mycobacterial systems, where doubling times are already slower than *E. coli*,^33^ careful synchronization of induction and growth phase is likely critical to achieving reproducible yields.

While our findings establish a robust proof-of-concept, several limitations must be acknowledged. First, the system was validated only with mCherry as a reporter. Future studies should assess larger or structurally complex antigens as well as proteins of biomedical interest from *M. tuberculosis*. Second, secretion efficiency and stability of exported proteins may vary depending on size, folding, and host processing. Finally, while riboswitches provide strong ON/OFF regulation, their dynamic range and sensitivity can be context-dependent, requiring further engineering for broad applicability.^34^

In conclusion, this study establishes translationally gated secretion as a new regulatory paradigm for protein production in mycobacteria. By enabling conditional access to the secretory machinery without reliance on protein-based regulators, this modular platform expands the synthetic biology toolkit available for mycobacterial research. Its ability to decouple transcription from secretion opens new possibilities for antigen screening, controlled production of conditionally toxic proteins, and functional analyses of proteins from slow-growing pathogenic species.

## 4 Methods

### 4.1 Construction of Riboswitch-Based Vectors for Controlled Expression and Secretion

The shuttle plasmid pUS976 was originally constructed in our laboratory as a mycobacterial expression and secretion vector. The backbone was derived from a ColE1-based *E. coli* replicon fused with a mycobacterial origin of replication, ensuring stable maintenance in both hosts. A kanamycin resistance cassette was included for dual selection in *E. coli* and mycobacteria. To enable constitutive expression and extracellular secretion, the promoter and signal peptide sequence of *M. tuberculosis* Ag85A were amplified by PCR and cloned upstream of a multiple cloning site. The fragment, containing the complete Ag85A promoter region and the N-terminal signal peptide coding sequence, was inserted into the backbone using BamHI and HindIII restriction sites, creating a transcriptional cassette that directs the expression and secretion of heterologous proteins. A polylinker was positioned downstream of the signal peptide to facilitate in-frame cloning of open reading frames, allowing translational fusion to the secretion signal. The construct was verified by Sanger sequencing, establishing pUS976 as a constitutive expression–secretion vector suitable for recombinant protein production in mycobacteria.

Riboswitch-based derivatives were generated by replacing the native Ag85A promoter–signal peptide cassette (hereafter termed the “native promoter”) with a recombinant cassette comprising the p85 promoter (without the 20 nucleotides upstream of the ATG, excising the native RBS), a riboswitch sequence, and the Ag85A signal peptide (hereafter termed the “recombinant promoter”). Three riboswitches were tested: riboE and riboE+ (both responsive to theophylline) and riboU9 (a temperature-sensitive thermoswitch). Recombinant promoters containing riboE or riboE+ were assembled by fusion PCR using PrimeSTAR GXL DNA polymerase (Takara Bio) according to the manufacturer’s instructions. In the first round, the individual fragments (p85, riboswitch, and signal peptide) were amplified with 10-nucleotide overlaps with adjacent sequences. In the second round, purified fragments were fused by using primers p85for and PSFbpArev. Riboswitch sequences were amplified from synthetic oligonucleotides: riboEE+5′ paired with riboE3′ (for riboE) or riboEE+5′ paired with riboE+3′ (for riboE+). The p85 promoter and signal peptide were amplified from pUS976 as a template. All primers and templates used are listed in Table 1.

**Table 1:** List and Sequences of the Oligonucleotide Primers Employed in This Study

name	sequence (5́-3́)	description
riboEE+5́	gaattcactagtatacgactcactataggtgataccagcatcgtcttgatgcccttgg	5́ template for riboE and riboE+
riboE3́	gaattccatcttgttgttacctccttagcagggtgctgccaagggcatcaagacgatg	3́ template for riboE
riboE+3́	gaattccatcttgttgcctccttagcagggtgctgccaagggcatcaagacgatg	3́ template for riboE+
p85for	aaaggatccccggctacatcga	forward primer for p85 amplification
p85rev(riboEE+)	gtatgcatgctctcaacgcatccatgcatg	reverse primer for p85 amplification
riboEE + for(p85)	tgcgttgagaactagtatacgactcactatag	forward primer for riboE and riboE + amplification
riboErev(PS)	caacaagctgcatcttgttgttacctcctta	reverse primer for riboE amplification
riboE + rev(PS)	caacaagctgcatcttgttgcctccttagc	reverse primer for riboE + amplification
PSFbpAfor(riboEE+)	caacaagatgcagcttgttgacagggtt	forward primer for signal peptide amplification
PSFbpArev	ttttctagacgggaaaatgcccc	reverse primer for signal peptide amplification
mcherry_RTfor	tcaacggccacgagttcgag	RT-qPCR primers for mcherry
mcherry_RTrev	aggccttgctgccgtacatg	
mysA_RTfor	gctgctgcaggacctgggcc	RT-qPCR primers for *mysA*
mysA_RTrev	agctggctgtcaccctcgtc	
mcherry_for	aaatctagaagcgatcatcaaggagttcatg	Primers for mcherry cloning
mcherry_rev	tttaagctttcacttgtacagctcgtccat	

PCR products were analyzed by 1% agarose gel electrophoresis and visualized with ethidium bromide under UV light. The recombinant promoter containing the U9 riboswitch was commercially synthesized (IDT) with BamHI and XbaI restriction sites at the flanks. After purification, both the recombinant promoters and pUS976 were digested with BamHI and XbaI (Anza, ThermoFisher), purified with the Wizard SV Gel and PCR Clean-up System (Promega), and ligated using T4 DNA ligase. The ligation mixtures were used to transform electrocompetent *E. coli* TOP10 cells. Recombinant plasmids were confirmed by colony PCR and Sanger sequencing. The reporter gene mCherry was then cloned into the XbaI and HindIII sites of all constructs (pUS976 and riboswitch variants). The gene was amplified from plasmid pCherry3 using primers mcherry_for and mcherry_rev. Verified constructs were subsequently transformed into electrocompetent *M*. smegmatis mc^2^ 155 cells. Transformants were selected on a Luria–Bertani (LB) agar supplemented with kanamycin (25 μg/mL).

For expression assays, recombinant *M. smegmatis* strains were cultured in LB medium containing 0.05% (v/v) Tween 80 and 25 μg/mL kanamycin (LB/Tw80/kan), with shaking at 200 rpm until saturation. Initial optimization determined the optimal theophylline concentration for riboE and riboE + constructs by monitoring secreted fluorescence across a gradient (0–5 mM). Maximal expression was obtained at 2 mM, which was used for all subsequent assays. Noninduced controls were maintained without theophylline, while induced cultures were supplemented with 2 mM. For riboU9 constructs, induction was achieved by shifting cultures from 20 to 25 °C or 37 °C. All riboE/E+ experiments were conducted at 37 °C. Samples were collected for RNA extraction (stabilized with TRIzol and stored at −80 °C), protein analysis (secreted and intracellular fractions for Western blot), and fluorimetric assays. For normalization, culture optical density at 600 nm (OD_600_) was recorded at each sampling point.

### 4.2 Detection of Reporter Gene Transcription by RT-PCR

RNA was extracted from TRIzol-preserved samples (Invitrogen). Bacterial cells were lysed by mechanical disruption with a Mini BeadBeater (Biospec Products Inc.) using zirconium beads. After clarification, chloroform was added for phase separation, followed by nucleic acid precipitation with 100% ethanol. Subsequent purification steps followed the Ribopure-Bacterial Kit protocol (Life Technologies). RNA quality and concentration were assessed by Nanodrop spectrophotometry (260/280 nm ratio), and samples were treated with Turbo DNase (Ambion) according to the manufacturer’s instructions.

cDNA synthesis was performed with the SuperScript III First-Strand Synthesis System (Invitrogen) by using random primers. Qualitative RT-PCR was then carried out to detect mCherry transcripts, with *mysA* (a constitutively expressed gene) serving as the normalization control (primers described in Table 1). PCR products were analyzed by 2% agarose gel electrophoresis.

### 4.3 Detection of Reporter Gene Expression by Western Blotting

Both secreted and intracellular protein fractions were analyzed via Western blotting. Culture aliquots (1.8 mL) were centrifuged at 16,000*g* for 10 min, and 1.6 mL of supernatant was carefully collected to avoid cell carryover. For intracellular analysis, the pellet was resuspended in lysis buffer (50 mM HEPES/KOH, pH 7.5; 10 mM MgCl_2_; 60 mM NH_4_Cl; 10% [v/v] glycerol) and lysed with a Mini BeadBeater (Biospec Products Inc.) using glass beads. Proteins from both the supernatant (secreted fraction) and lysate (intracellular fraction) were precipitated with 100% trichloroacetic acid (final concentration 17%). Precipitates were washed with ice-cold acetone containing 1% triethanolamine (TEA).

Secreted proteins were resuspended in 30 μL of SDS-PAGE sample buffer (100 mM Tris–HCl, pH 6.8; 4% SDS; 20% glycerol; 0.2% bromophenol blue; 200 mM β-mercaptoethanol), while intracellular proteins were resuspended in buffer normalized to 0.2 OD_600_ per 10 μL. All samples were boiled at 100 °C for 5 min before electrophoresis.

Proteins were resolved on 12% resolving and 4% stacking SDS-PAGE gels and transferred onto nitrocellulose membranes using the Bio-Rad Trans-Blot Semi-Dry system (100 V, 1 h). Transfer efficiency was verified by reversible staining (MEM Code Stain, Thermo Scientific). Membranes were blocked with 10% skim milk in Tris-buffered saline (TBS).

Blots were incubated for 2 h with primary antibody (polyclonal anti-mCherry, Invitrogen PA534974; 1:1000 in 5% milk/TBS), washed three times with TBS-T, and rinsed with TBS. The secondary antibody (goat antirabbit IgG [H + L], HRP-conjugated; Thermo Fisher Scientific) was applied at 1:10,000 dilution for 1 h under the same conditions. After washing, detection was performed using the SuperSignal West Pico Chemiluminescent Substrate kit according to the manufacturer’s protocol.

### 4.4 Detection of the Reporter Gene Function by Fluorimetric Assay

mCherry fluorescence was quantified from both secreted and intracellular fractions. Culture aliquots (1.0 mL) were centrifuged at 16,000*g* for 10 min to separate the supernatant (secreted fraction) from the pellet (intracellular fraction). For secreted protein analysis, 200 μL of the supernatant was transferred to a black 96-well clear-bottom plate (Corning), with LB/Tw80/kan medium as the blank. For intracellular analysis, pellets were washed three times with 1× PBS, resuspended to an OD_600_ of 0.5, and 200 μL of suspension was loaded per well using PBS as the blank. Fluorescence was measured with a Varioskan Lux microplate reader (excitation: 587 nm; emission: 610 nm; bandwidth: 10 nm). Data were expressed as relative fluorescence units (RFU), calculated as the ratio of the fluorescence signal to the OD_600_ of the culture at sampling. All experiments were performed in biological triplicates. Data are presented as mean ± standard deviation, and statistical significance was assessed by one-way ANOVA (GraphPad Prism 5).

### 4.5 Analysis of the Optimal Growth Stage for Inducing Expression in Riboswitch-Based Systems

Optimization of induction timing was evaluated at the early-, mid-, and late-logarithmic phases. For riboE and riboE + constructs, cultures were grown at 37 °C and induced with 2 mM theophylline. For riboU9, cultures were maintained at 20 °C during growth and induced by a temperature shift to 37 °C. All experiments were performed in LB/Tw80/kan medium with an initial OD_600_ of 0.05.

For riboE/E+ constructs, early-, mid-, and late-log phases were reached at approximately 10 h (OD_600_ ≈0.4–0.5), 18 h (OD_600_ ≈1.9–2.0), and 24 h (OD_600_ ≈2.7–2.8), respectively. For riboU9 constructs, growth at 20 °C prolonged doubling times, with phases reached at 16 h (early-log), 22 h (mid-log), and 30 h (late-log). Following induction, aliquots were collected hourly to measure the fluorescence in the secreted fraction.
